# Vignetting and Field of View with the KAMRA Corneal Inlay

**DOI:** 10.1155/2013/154593

**Published:** 2013-11-13

**Authors:** Achim Langenbucher, Susanne Goebels, Nóra Szentmáry, Berthold Seitz, Timo Eppig

**Affiliations:** ^1^Experimental Ophthalmology, Saarland University, Kirrberger Strasse 100, Building 22, 66424 Homburg, Germany; ^2^Erlangen Graduate School in Advanced Optical Technologies (SAOT), 91052 Erlangen, Germany; ^3^Department of Ophthalmology and University Eye Clinic, Saarland University Medical Center, 66424 Homburg, Germany

## Abstract

*Purpose*. To evaluate the effect of the KAMRA corneal inlay on the retinal image brightness in the peripheral visual field. *Methods*. A KAMRA inlay was “implanted” into a theoretical eye model in a corneal depth of 200 microns. Corneal radius was varied to a steep, normal, and flat (7.37, 7.77, and 8.17 mm) version keeping the proportion of anterior to posterior radius constant. Pupil size was varied from 2.0 to 5.0 mm. Image brightness was determined for field angles from −70° to 70° with and without KAMRA and proportion of light attenuation was recorded. *Results*. In our parameter space, the attenuation in brightness ranges in between 0 and 60%. The attenuation in brightness is not affected by corneal shape. For large field angles where the incident ray bundle is passing through the peripheral cornea, brightness is not affected. For combinations of small pupil sizes (2.0 and 2.5 mm) and field angles of 20–40°, up to 60% of light may be blocked with the KAMRA. *Conclusion*. For combinations of pupil sizes and field angles, the attenuation of image brightness reaches levels up to 60%. Our theoretical findings have to be clinically validated with detailed investigation of this vignetting effect.

## 1. Introduction

Physiological accommodation is well known to decrease over time and to end-up in presbyopia, a condition where accommodation is no longer sufficient for focusing on objects at near distance. It has been a dream for ophthalmic surgeons for a very long time to recover accommodation in progressed age, and many attempts have been made to develop active or passive lens implants (such as accommodating lenses) [1–6], refractive or diffractive multifocal lenses [7], customized photorefractive keratectomy (PRK) or Laser in situ keratomileusis (LASIK) ablations using the excimer laser generating sectorial or zonal near focus region in the cornea [8–11], or changing the global shape of the cornea to a hyperprolate surface [8, 12].

Most of those options for overcoming presbyopia have serious drawbacks: active accommodating lenses require energy buffers in or adjacent to the eye and they may be interfering with electrical or magnetic fields (e.g., during MR examination). Passive accommodative lenses today are mostly designed as translation lenses with one or more optics and may lack sufficient accommodation [4, 13, 14] in case of lens epithelial cell proliferation (e.g., with secondary cataract); multifocal lenses show strong deteriorations in contrast transmission due to superposition of images in focus and out of focus and straylight and customized excimer laser ablations are often subject to regression effects and irregular astigmatism in the transient zone between near and far distance focus.

One of the latest developments addressing presbyopia is the (intra)corneal pinhole inlay currently marketed under the name KAMRA (previously AcuFocus, AcuFocus Inc., Irvine, USA) [15]. This inlay is a ring-shaped aperture stop which is using the pinhole effect for smearing the focus in longitudinal direction increasing the depth of focus (DOF). This inlay is made of a thin tinted film layer (biocompatible polymer) and installed in the anterior cornea of the nondominating eye after generating a flat bag using a femtosecond laser [16]. Centration of the corneal inlay is crucial [17, 18]. For coaxial light, in small pupil sizes, all rays are passing through the central hole of the aperture, but in larger pupil sizes rays are also passing through the peripheral cornea outside the edge of the inlay. Other types of corneal inlays available for presbyopia correction are based on different principles of action. These corneal inlays act as bi- or multifocal lens such as the Presbia Flexivue Microlens (Presbia, Irvine, CA, USA) [19] or alter the shape of the anterior corneal surface such as the Vue+ or Raindrop lens (ReVision Optics, Inc., Lake Forest, CA, USA) [20].

Up to now, the effect of the KAMRA inlay is only described in theoretical and clinical studies, which proof the effect of recovering proper results for far and near distance visual function [16–18, 21–25]. Pepose recently reported results for binocular mesopic and photopic contrast vision after monocular KAMRA implantation, which remained unchanged for distance vision but improved significantly for near vision [26]. Long-term results are very limited [24]. Decentration effects and theoretical image quality have been investigated already [18] but, up to our knowledge, there is no work done on the reduction of light passing through the eye after implantation of a KAMRA and about vignetting effects as a function of field of view. Vignetting is the effect of reduced image brightness in the periphery or other parts of the image. This is known from photography and sometimes used to beautify artwork.

The purpose of our study was to simulate the effect of image brightness in a schematic model eye after implantation of a KAMRA inlay in comparison to the respective model eye without KAMRA for variations of corneal radius of curvature and proportions of pupil size to anterior chamber depth as a function of field angle (vignetting effect).

## 2. Methods

For our simulation, we used a modified Liou-Brennan schematic model eye (LBME) [27]. Therefore, we adopted the Liou-Brennan model eye used in our previous studies [28–30] including the decentered pupil. There are various model eyes available for optical simulation which can be customized to individual biometric properties [31].

**Figure 1:**
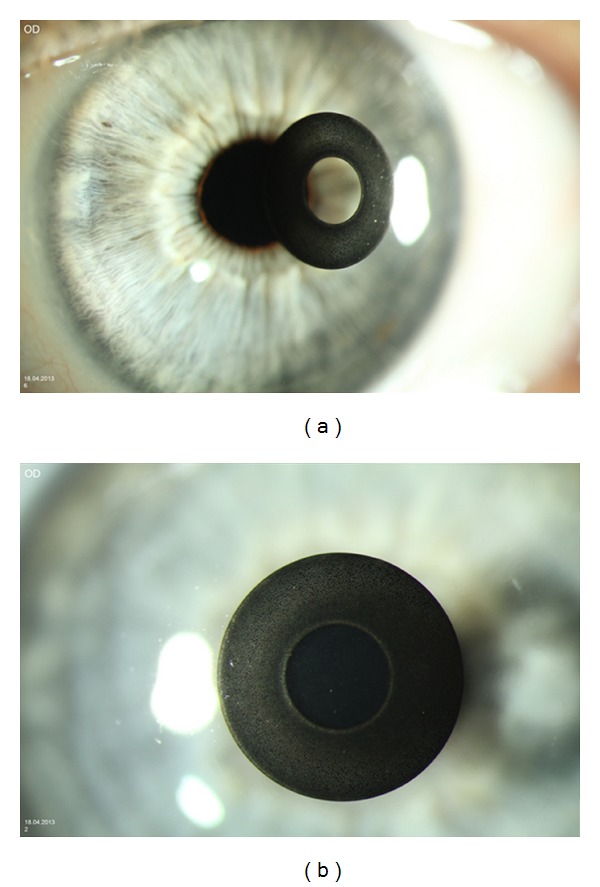
Photograph of a KAMRA inlay in a patient eye (a). The inner diameter is 1.6 mm which acts as a pinhole. The outer diameter is 3.8 mm. This thin film layer (5 microns) shows thousands of tiny randomly distributed perforations ensuring nourishment of the corneal tissue (b).

A thin diaphragm (aperture stop) was placed in a distance of 200 microns behind the corneal surface (virtual flap generation for KAMRA implantation). The inner diameter of the KAMRA is 1.6 mm and the outer diameter is 3.8 mm (Figure 1 shows a KAMRA implanted in a patient eye). With a thickness of 5 microns, we assumed that there is no effect on the corneal shape due to the implantation of the inlay. The thousands of randomly distributed laser drilled tiny perforations are made for ensuring nutrition of the corneal tissue and do not participate in the optical effect (beside a negligible reduction of the contrast due to straylight). The KAMRA inlay was centered to the visual axis (line of sight) of the model eye, which is tilted 5° in respect to the optical axis [27]. The (internal) anterior chamber depth (ACD) was kept constant in our model at 3.16 mm and the pupil size was changed from 2.0 mm to 5.0 mm in steps of 0.5 mm. As the magnification of the cornea has to be considered, this refers to a visible pupil size of 2.4 mm to 6.0 mm in steps of 0.6 mm.

**Figure 2:**
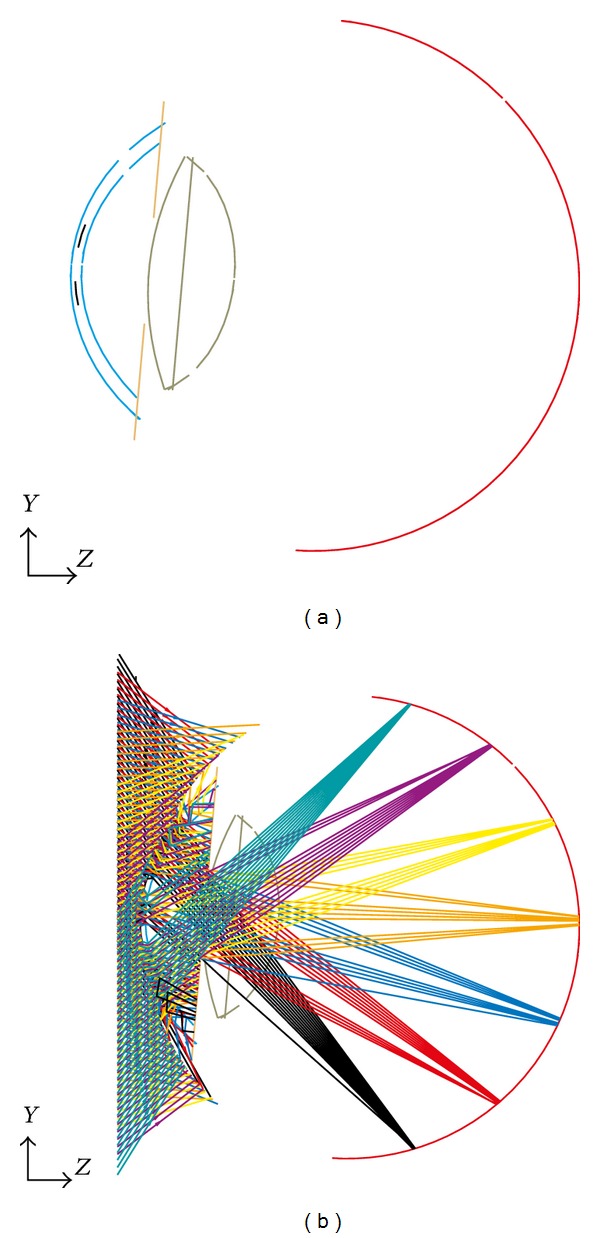
(a) Cross section of the Liou-Brennan schematic model eye created in ASAP with a KAMRA inlay (black) within the cornea (blue), the iris (orange), the crystalline lens (olive), and the retina (red). (b) Cross section of the model eyes including traced ray bundles in different colors which refer to different field angles (shown from −60° to +60° in steps of 20°). The effect of this additional aperture stop is directly visible.

For our modeling, we used the optical simulation software ASAP (Version 2006 V1R1, Breault Research Organization, Tucson, USA), and slightly diverging rays emerging from a virtual hemispherical surface with 0.2 m radius around the cornea were traced through the system. This situation was chosen to imitate a perimeter hemisphere. We created 280 sources on the hemisphere at visual field angles of −70° (temporal) to 70° (nasal) in steps of 0.5°. Each source was defined by 100 rays with uniform intensity (Figure 2). The Stiles Crawford effect was implemented as apodization function in the entrance pupil with a Gaussian approximation at 3.4 mm pupil radius (1/*e* ^2^ intensity). This value was derived from the approximation *L*_*e*_(*r*) = exp⁡(−*βr* ^2^) with *β* = −0.173 which covers 97.6 percent of the population [32].

The simulation was restricted to a monochromatic situation at a wavelength of *λ* = 546 nm. The respective model eye without KAMRA was used as reference. Figure 2 shows the principle situation with the KAMRA with a reduced number of ray fans entering the eye.

We calculated the intensity distribution at retinal plane which allows us to directly evaluate the regions in the fields of view which are affected by attenuation of the KAMRA. We then calculated the intensity of the eye models with KAMRA in relation to the intensity without KAMRA (intensity attenuation).

## 3. Results

The KAMRA was curved in a way that it was for all corneal shapes parallel to the corneal front surface, which is in accordance with the flap generation technique of common femtosecond lasers, as the KAMRA is usually implanted into a pocket or under a flap generated with femtosecond laser technology. As the corneal thickness was kept constant at a value described in the Liou-Brennan model eye for all corneal shapes, the distance of the KAMRA inlay to the corneal back surface (equal to residual stromal bed) was 295 microns.

As we varied the pupil size by keeping the (internal) anterior chamber depth of the eye constant at the value described by the Liou-Brennan model eye (3.16 mm), the ratio of pupil size to anterior chamber depth (aspect ratio) was 0.63, 0.79, 0.95, 1.07, 1.27, 1.42, and 1.58 for pupil sizes 2.0, 2.5, 3.0, 3.5, 4.0, 4.5, and 5.0 mm.

**Figure 3:**
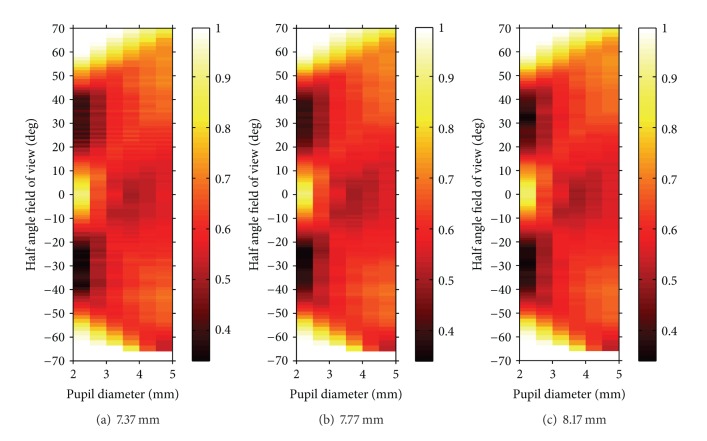
Relative illumination at the retina for variation of pupil size and field angle with the Liou-Brennan model eye without KAMRA exemplarily for variations of pupil diameter and different corneal front surface radii of  7.37,  7.77, and 8.17 mm in subfigures (a)–(c), respectively.

Figure 3 shows the relative illumination at the retina for variation of pupil size and field angle with the Liou-Brennan model eye without KAMRA inlay exemplarily for a corneal front surface radius of 7.37 mm (Figure 3(a)), 7.77 mm (Figure 3(b)), and 8.17 mm (Figure 3(c)).

**Figure 4:**
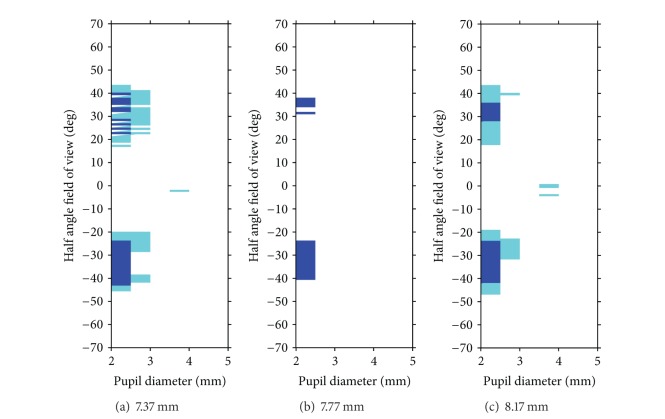
Regions in the visual field with more than 50% (light blue) or 60% (dark blue) of attenuation for variations of pupil diameter and different corneal front surface radii of  7.37,  7.77, and 8.17 mm in subfigures (a)–(c), respectively.

Figure 4 displays the areas of more than 50% or more than 60% attenuation with KAMRA to the situation without KAMRA inlay for a corneal front surface radius of 7.37 mm (Figure 4(a)), 7.77 mm (Figure 4(b)), and 8.17 mm (Figure 4(c)). This graph shows that, especially with small pupil sizes, the attenuation becomes relevant in the midperipheral visual field of 20° to 40°.

## 4. Discussion

Nowadays, KAMRA inlays are very popular as a treatment option for presbyopia [15–17, 21–26, 33]. The manufacturer as well as key opinion leaders propagates the KAMRA as an effective tool to overcome loss of near vision with age. They postulate that there are more or less no adverse effects and the treatment can be reversed by explanting the inlay [34]. Only one report about the complications after implantation of a KAMRA in a rabbit eye is available [35]. First clinical publications on that topic show that near vision can be improved significantly [17, 21, 22, 24] and the defocus curve could be broadened comparable to the situation with multifocal lenses. On the other hand side, there are reports that the central visual field is not affected with implantation of those inlays [23, 26], but detailed clinical data has not been published up to now. Especially, the mid-to-peripheral visual field may be affected by a KAMRA, as it has been shown for other aperture restricting optical implants such as keratoprostheses [36]. Therefore, in the present paper, we address the effect of light attenuation for variations of field angle and pupil size in an optical simulation model.

A corneal pinhole inlay is acting in the eye as a second aperture stop. Beside the pupil of the eye, the KAMRA implanted in the anterior part of the cornea is a ring-shaped aperture with a pinhole of 1.6 mm and an outer diameter of 3.8 mm (Figure 1). If the inlay is properly centered and light is entering coaxially (field angle 0°), only the central pinhole is relevant if the pupil size is less than approximately 4.56 mm (with pupil magnification of 1.2 [37]). If the pupil size is becoming larger, rays are also passing outside the edge of the KAMRA. For field angles unequal zero, such simple thoughts cannot be performed and ray tracing techniques are required to tailor out which rays are blocked by the KAMRA or the pupil of the eye.

We simulated both situations—with and without KAMRA—with professional optical design software on a modern schematic model eye. A bundle of rays was projected to the cornea and we counted the number of rays which were passing through the pupil (in the Liou-Brennan eye) or the KAMRA and the pupil (Liou-Brennan eye with KAMRA). To keep the model simple, we ignored the effect of variation of corneal thickness, depth of the layer where the KAMRA is implanted, anterior chamber depth or shape, and optical properties of the crystalline lens. In contrast, we addressed the effect of corneal shape, pupil size, and field angle of an object, which has to be imaged to the retina. The KAMRA was aligned properly to the visual axis and we ignored decentration effects in our simulation. However, these may have a significant effect on the performance of this presbyopia treatment option. The importance of proper centration and residual ametropia for the visual results with the KAMRA have been investigated by Artal et al. [18, 38].

We found out that the effect of the corneal shape on the brightness attenuation is negligible; however, the ratio between anterior chamber depth and pupil diameter may play a more important role. Especially in hyperopic patients, the ratio between pupil diameter and anterior chamber depth may become small so that the attenuating effect of the KAMRA may be even more significant. For large field angles where the incident ray bundle is passing through the peripheral cornea just missing the KAMRA retinal image, brightness is not affected. For small field angles there is a significant attenuation in brightness, and the worst case scenario is a combination of small half field angles (0–3°) and pupil sizes of 3.0 or 3.5 mm or small pupil sizes (2.0 and 2.5 mm) and field angles of 20–40°. In those situations, the KAMRA is blocking out most of the light.

This is in full accordance with what we expected: for a field angle of 0° (coaxial illumination), the brightness at the retina remains unchanged if the pupil is getting larger from 1.6 × 1.2 = 1.92 mm to 3.8 × 1.2 = 4.56 mm (simplifying the pupil magnification by a factor of 1.2). If the pupil size is becoming larger, light is passing at the outer edge of the KAMRA increasing retinal illumination. However, this is inadvertently accompanied by an increase in aberrations and therefore a decrease in contrast sensitivity; on the other hand, this effect is counteracted by an increase of retinal sensitivity. For small pupil sizes (e.g., 2.0 mm) and half field angles of 20–40°, the rays which would pass through the pupil are blocked by one side of the ring aperture. This may have different effects with previously hyperopic or myopic patients which had undergone refractive surgery before KAMRA implantation as KAMRA implantation is currently only suggested for eyes with emmetropic or slightly myopic refraction [18].

The potential clinical consequences may be that the attenuation of the brightness is affecting the visual field at the nondominant eye, where the KAMRA is implanted. These simulation results have to be verified in the future by clinical measurements testing larger visual fields.

Figure 4 shows the combinations of pupil sizes and field angles, where the KAMRA is reducing the light passing through the retina to an extent of 50% or more (for corneal front surface radii of 7.37, 7.77, and 8.17 mm). Those combinations of parameters have to be addressed when the results of this simulation study are manifested with clinical data, which is not the scope of the present work.

The pupil function is well known to be linked between both eyes [39–41]. With a light stimulus at one eye, the pupils of both eyes are reacting irrespective whether the stimulus is applied at the dominant or the nondominant eye. This is important, because the light stimulus at the nondominant eye, where the KAMRA is implanted, is attenuated significantly by the aperture function of the KAMRA and the attenuation depends on the field angle.

Future research should address the effects of corneal asphericity and preoperative refraction on the performance of the KAMRA inlay and the combination with intraocular lenses.

In conclusion, we performed an optical simulation on a new treatment option for correcting presbyopia, the corneal pinhole inlay. We found that for combinations of pupil sizes and field angles the attenuation of image brightness may reach levels of more than 60% causing potential loss of contrast sensitivity, which seems to be clinically relevant from our point of view. Further studies have to be performed which validate our simulation results in a clinical setup and which address the clinical consequences of this vignetting effect more in detail.
